# Comparative Efficacy of Different Oral Doses of Clindamycin in Preventing Post-Operative Sequelae of Lower Third Molar Surgery—A Randomized, Triple-Blind Study

**DOI:** 10.3390/medicina58050668

**Published:** 2022-05-17

**Authors:** Anna Janas-Naze, Gaja Torbicka, Damian Chybicki, Małgorzata Lipczyńska-Lewandowska, Wei Zhang

**Affiliations:** 1 Department of Oral Surgery, Medical University of Lodz, Pomorska 251, 92-213 Lodz, Poland; g.torbicka@gmail.com (G.T.); damian.chybicki@gmail.com (D.C.); malgorzata.lipczynska-lewandowska@stud.umed.lodz.pl (M.L.-L.); 2 Shanxi Oral Disease Prevention and Treatment Center, Shanxi Provincial People’s Hospital, Taiyuan 030012, China; wei_zhang8821@163.com

**Keywords:** clindamycin, dosage, third molar extraction, disimpaction, randomized controlled trial, oral drug delivery

## Abstract

*Background and Objectives*. Antibiotic regimen optimization is a major concern in post extraction sequelae management following third molar surgery, mostly owing to the absence of universal guidelines. Hence, this study aimed to determine the effect of antibiotic prophylaxis using three different doses of clindamycin on the prevention of infection and other complications following mandibular third molar disimpaction. The secondary outcome was testing whether clindamycin exhibits activity in acute or chronic models of pain using the visual analog scale of pain and the necessity for post-operative rescue analgesia. The tertiary outcome was to assess clindamycin penetration into the saliva by measuring its concentration using liquid chromatography/electrospray ionization tandem mass spectrometry. *Materials and Methods*. A randomized, two-center, triple-blind, controlled clinical trial was conducted, in which the patients were randomly allocated to three groups: I, receiving 150 mg clindamycin every 8 h; II, receiving 300 mg clindamycin every 8 h; and III, receiving 600 mg clindamycin every 12 h. Each group continued the therapy for five days. *Results*. An overall decrease in the risk of infection and other post-operative complications, such as trismus, edema, dysphagia, and lymphadenopathy, was achieved, with the best results in group I. *Conclusion*. As no statistical association was observed between clindamycin concentration in saliva and degree of post-operative inflammation, clindamycin concentration, or pain intensity, smaller doses of clindamycin administered over shorter time periods is recommended.

## 1. Introduction

Most oral surgeons recommend the administration of antibiotics following third molar surgery; however, despite various studies, no consistent guidelines on the optimization of antibiotic regimens exist; thus, prophylactic antibiotic therapy remains debatable [1,2]. Certain studies [3,4] have indicated the benefits of routine prophylaxis, while others recommend its use only in severe cases [5,6], since excessive prescription may result in antibiotic resistance. Administered drugs should be active against the anticipated pathogens and reach sufficient tissue and fluid concentrations for effective action. 

Since surgical extraction for impacted mandibular third molars remains the most common procedure in the field of oral surgery, post-operative complications are anticipated to pose a clinical challenge. Although current surgical techniques allow for aseptic procedures and handling of tissues, post-operative infections still occur. 

The rate of infection following surgical extraction of impacted mandibular third molars varies from 0 to 16% [7] based on the type of procedure, presence of infection, duration of surgery, and the systemic condition of the patient. Its severity escalates with the development of assorted aerobic and anaerobic bacterial flora, Gram-positive cocci and Gram-negative bacilli being the primary anaerobic microorganisms. However, these infections initially manifest as aerobic bacteria-supported cellulitis [8], with major isolates belonging to the streptococci group. Subsequent tissue degradation with possible abscess formation results, owing to proteolytic enzymes, endotoxins, and exotoxins produced by anaerobic bacteria, mostly *Peptostreptococcus*, *Veillonella*, *Prevotella*, and *Actinomyces* species. 

Since the results of microbiological examination are rarely available in a short time frame owing to the lengthy incubation process, confirmation of the exact microorganisms and their antimicrobial susceptibility is seldom obtained. The polymicrobial nature of odontogenic infections requires the use of pharmaceuticals that show activity towards both anaerobic and aerobic bacteria; hence, clinicians decide between penicillin, amoxicillin, and clindamycin administration. 

Clindamycin, a lincosamide antibiotic, is an effective antimicrobial agent that is used in the treatment of specific mixed-flora infections. It is mostly absorbed upon oral administration, with peak concentrations within one hour, and concomitant administration of food does not visibly modify serum concentrations [9]. It has also been proven that even at doses below the minimal inhibitory concentration (MIC), the use of clindamycin may result in inhibition of the synthesis of bacterial toxins [10], which is exceptionally significant from a clinical point of view since it limits the adverse effects of bacterial toxins even before the antibiotic impedes the actual proliferation of bacteria. Another study [11] further demonstrated that clindamycin was able to retard the synthesis of streptolysin S and M, produced by *Streptococcus pyogenes*, a significant majority of exotoxins produced by *Staphylococcus aureus* (i.e., alpha-hemolysin or protease), and extracellular lipase produced by *Propionibacterium acnes* and *granulosum*. A decrease in exotoxin synthesis results in reduced bacterial virulence, ultimately facilitating the elimination of microorganisms by the immune system. 

Clindamycin is the antibiotic of choice in patients with penicillin hypersensitivity [12] and its clinical value is well known; however, amoxicillin is preferred to clindamycin following third molar surgery. Our department has been using clindamycin to prevent the sequelae of third molar surgeries for several years with good results, which piqued our interest in comparing different doses of this drug in similar settings so as to determine whether smaller doses are as effective in preventing adverse events following third molar surgery as higher ones, regardless of patient variables. This topic seems particularly important, since previous research [13] clearly indicates that the decision to prescribe prophylactic antibiotics to prevent post-operative complications after oral surgery procedures is mostly based on the subjective considerations of clinicians. Another study [14] demonstrated that odontogenic infections account for as much as 10% of overall antibiotic usage each year, despite clear inadequacy in the translation of evidence-based information into clinical practice, as is proven especially by inconsistencies regarding the prescription, dosages, and durations of antibiotic courses.

Hence, this study aimed to determine the effect of antibiotic prophylaxis on the prevention of infection and other complications following surgical extraction of impacted mandibular third molars using three different doses of clindamycin hydrochloride. The secondary outcome was testing whether clindamycin exhibits activity in acute or chronic pain models using the visual analog scale (VAS) of pain and evaluating the need for post-operative rescue analgesia. The tertiary outcome was to assess the penetration of clindamycin into saliva by measuring the clindamycin concentration using reverse-phase high-performance liquid chromatography tandem mass spectrometry (LC/ESI-MS/MS).

## 2. Materials and Methods

### 2.1. Study Design

A triple-blind, parallel, randomized, two-center clinical trial was conducted in the Department of Oral Surgery, Medical University of Lodz, Poland, and the Shanxi Oral Disease Prevention and Treatment Center, Shanxi Provincial People’s Hospital, China, over a period of 12 months from February 2021 to February 2022. The study was approved by the Bioethical Committee of the Medical University of Lodz and the Medical Ethics Committee of the Shanxi Provincial People’s Hospital, in compliance with the Helsinki Declaration, following the acquisition of informed consent from the participants. 

In order to ensure the highest possible reproducibility of the results, only patients with mandibular third molars with complete root development, retained vertically or mesioangularly (according to Winter’s classification), and belonging to class II B or C (Pell and Gregory classification), and extracted for orthodontic reasons, were included in this study.

Since the study followed a triple-blind, randomized protocol, the patient, evaluator, and laboratory personnel were blinded to the group assignment. The surgeon, who was not involved in the evaluation process, was responsible for randomization, delivery, and guidance concerning the administration of the drugs. Randomization was performed using Research Randomizer (randomizer.org), with an allocation ratio of 1:1:1, and the code was unknown to the rest of the investigators until the collection of clinical data was terminated. A total of 285 blocks, with three random numbers in each block (corresponding to the three dose regimens), were generated. These random numbers were categorized into sequences of 1–95 for 150 mg (I), 95–190 for 300 mg (II), and 190–285 for 600 mg (III) of clindamycin. The sealed envelopes were labeled with appropriate codes and included in the administration protocol. Following surgery and randomization, the patient received two sealed boxes, one containing clindamycin and the rescue drug (400 mg ibuprofen, US Pharmacia, Anhui Medipharm, 4 capsules). Identical, non-marked capsules containing 150, 300, or 600 mg of clindamycin hydrochloride were prepared and coded in the hospital pharmacy. Patients were randomly allocated to three groups: I, receiving 150 mg clindamycin every 8 h; II, receiving 300 mg clindamycin every 8 h; and III, receiving 600 mg clindamycin every 12 h after surgery for five days. 

### 2.2. Patient Selection

The inclusion criteria were as follows:

Child-bearing potential or non-child-bearing potential, female or male, non-smoker, ≥18 and ≤65 years of age at the time of signing the informed consent, weighing >50 kg.

Non-child-bearing potential female participant was defined as follows:

Post-menopausal state: absence of menses for 12 months prior to drug administration or history of hysterectomy with bilateral oophorectomy at least 6 months prior to drug administration;

Sterile hysterectomy, bilateral oophorectomy, or tubal ligation performed at least 6 months prior to drug administration;

Capable of consent;

Patients scheduled to undergo disimpaction of bony impacted mandibular molars under short-acting local anesthetic (e.g., mepivacaine, articaine, or lidocaine) preoperatively for orthodontic reasons;

Patients who signed a written informed consent form prior to any study-related procedures and who were able to understand and comply with the protocol requirements and instructions;

Female patients of childbearing potential willing to use a highly efficient birth control method during the study.

The following exclusion criteria were applied:

A history of clinically significant illnesses or surgeries within four weeks prior to the administration of the study medication;

Any clinically significant abnormality detected during the medical screening;

Any reason preventing the participation of the individual in the study ascertained by the investigator;

Positive testing for hepatitis B, hepatitis C, or human immunodeficiency virus upon screening;

Electrocardiographic abnormalities (clinically significant) or vital sign abnormalities (systolic blood pressure <90 over 140 mmHg, diastolic blood pressure <50 or >90 mmHg, or heart rate less than 50 or >100 bpm) upon screening;

BMI ≥ 30.0 kg/m^2^;

History of significant alcohol abuse within six months prior to screening or any indication of habitual consumption of more than 14 units of alcohol per week or a positive alcohol breath test upon screening. Alcohol abuse was defined as the consumption of more than 90 mL of liquor or spirit or 530 mL of beer per day for consecutive days during the 6-month period;

History of drug abuse or use of illegal drugs. Drug abuse was defined as any recreational drug use for five consecutive days during the 6-month period;

Patients with any known history of hypersensitivity to clindamycin or other related drugs (e.g., lincomycin, paracetamol, ibuprofen) and hypersensitivity reactions, including symptoms of asthma, rhinitis, angioedema, or urticaria;

History of heparin allergy;

Use of any drugs known to induce or inhibit hepatic drug metabolism (e.g., inducers, such as barbiturates, carbamazepine, phenytoin, glucocorticoids, rifampin/rifabutin; inhibitors, such as antidepressants, cimetidine, diltiazem, erythromycin, ketoconazole, monoamine oxidase inhibitors, neuroleptics, verapamil, and quinidine) within 30 days prior to the administration of the study medication;

Patients with a current or chronic history of liver disease, known hepatic or biliary abnormalities, severe renal impairment, or severe heart failure (New York Heart Association class IV);

Patients with a history of gastrointestinal bleeding or perforation related to previous antibiotic or nonsteroidal anti-inflammatory drug (NSAID) therapy, gastrointestinal hemorrhage, cerebrovascular hemorrhage, or other progressive hemorrhage, such as an active or history of recurrent peptic ulcer/hemorrhage (two or more distinct episodes of proven ulceration or bleeding);

Patients with inflammation or ulcerative disease of the oral mucosa;

Patients with known systemic lupus erythematosus;

Indication for extraction of contralateral molar in the same procedure;

Participants with very long trans-operative times (>60 min);

Patients treated with antibiotics, analgesics, or NSAIDs within 3 days prior to the day of randomization (or within 5 times the elimination half-life, whichever was considered longer);

Patients who received other antibiotics or analgesics rather than short-acting preoperative or intraoperative local anesthetic agents within 12 h before the start of the surgery or peri-operatively until randomization;

Patients with any current dental or medical condition preventing safe participation in this study;

Unwillingness or inability to follow the procedures outlined in the protocol;

Difficulty in swallowing the medication used in the study;

Use of any tobacco products 90 days prior to drug administration;

Participants with a clinically significant history of tuberculosis, epilepsy, asthma, diabetes, psychosis, or glaucoma;

Clinically significant history of diarrhea subsequent to the administration of antibacterial agents or antibiotics.

Additional exclusion criteria for females only:

Breast-feeding participants;

Positive urine pregnancy test at screening;

Female participants of childbearing potential engaging in unprotected sexual intercourse with any non-sterile male partner (i.e., a male who had not been sterilized by vasectomy for at least 6 months) within 14 days prior to study drug administration. 

### 2.3. Intervention

All surgeries were performed by two dental surgeons specializing in oral and maxillofacial surgery in the same manner. After achieving inferior alveolar and buccal nerve block, surgery was initiated by recording its duration (from the first incision to the last suture). Each surgical procedure consisted of designing and preparing a triangular mucoperiosteal flap, followed by osteotomy with saline irrigation. Following the extraction, the wound was sutured after achieving hemostasis. Post-operative written instructions were clearly provided and were repeated audibly. Patients were instructed to note the exact time of taking the rescue drug if the situation should arise and instructed not to use any other drugs during the trial. In cases of severe post-operative infection, patients were withdrawn from the study and treated adequately. 

Each patient was scheduled for follow-up on the first, third, and seventh days post-operation. 

### 2.4. Post-Operative Inflammatory Parameters

The following parameters were evaluated: trismus, facial edema, lymphadenopathy (submandibular lymph nodes), body temperature, local infection, and dysphagia.

Trismus, facial edema, and lymphadenopathy evaluations were performed using a 3-grade scale. 

Trismus was based on the classification previously used by Kaczmarzyk et al. [15]:

Grade 0—possibility of vertical insertion of three of the investigator’s fingers between the dental arches in the maxillary midline;

Grade 1—only two fingers;

Grade 2—only one finger;

Grade 3—inability of inserting even one finger.

Facial edema [15]:

Grade 0—no visible asymmetry;

Grade 1—slight swelling (increased cohesion of soft tissues with practically invisible asymmetry);

Grade 2—medium swelling (visible asymmetry);

Grade 3—significant swelling.

Lymphadenopathy:

Grade 0—lymph nodes not palpable;

Grade 1—slight enlargement (not painful);

Grade 2—enlargement (painful, movable);

Grade 3—substantial enlargement (highly painful).

Dysphagia grade was based on the classification defined by Saeed et al. [16]:

Grade 0—unable to swallow;

Grade 1—swallows liquids with difficulty but cannot swallow solids;

Grade 2—swallows liquids with no difficulty but cannot swallow solids;

Grade 3—occasional difficulty in swallowing solids;

Grade 4—rarely has difficulty in swallowing, only with solids;

Grade 5—swallows normally.

Temperature was considered notable if it exceeded 37.5 °C, taking into consideration the mechanisms of psychological stress. Local infection, defined as a dry socket, was diagnosed based on the clinical findings (premature loss of the alveolar clot, foul odor, and pain) and evaluated as presence (1) or absence (0).

### 2.5. Post-Operative Pain

To address the second objective, post-operative pain relief was compared among the three groups. The VAS was used to evaluate the intensity of pain experienced following surgery with respect to the dose of clindamycin. The VAS is a horizontal line with an eleven-point numeric range. It is labelled from zero to 10, with zero indicating no pain and 10 indicating the worst pain possible; therefore, a higher VAS score represents a worse outcome. Patients assessed their pain using the VAS once, 60 min after surgery, and three times afterwards (at 24 h, 72 h, and 7 days following the surgery).

### 2.6. Adverse Effects

All the adverse events, such as (but not limited to) nausea, vomiting, diarrhea, skin rash, itching, chest pain, abdominal or stomach cramps, pain or tenderness, were self-reported by the patients.

### 2.7. Clindamycin Concentrations in the Saliva

On days 1, 3, and 7 of clindamycin administration, parotid saliva samples were obtained from the parotid duct using a 22-gauge plastic catheter attached to 1 mL insulin syringes. The samples were then transferred to Eppendorf tubes and stored at <20 °C. All the samples were frozen until the assay. To perform a quantitative analysis of clindamycin in human saliva, an LC/ESI-MS/MS method developed by Catena et al. [17] was used. Separation was carried out at 50° on a rapid resolution, 30 mm × 2.1 mm column packed with C_18_ 3.5 µg silica reversed particles (Sigma-Aldrich, St. Louis, MO, USA). The mobile phase was a mixture of methanol–trifluoroacetic acid 0.01% (40:60, *v*/*v*). Separation was achieved using isocratic solvent elution at a flow rate of 0.6 mL/min. Quantitation was performed in multiple reaction monitoring mode to monitor the precursor-to-product ion transition of *m*/*z* 425.1 → 126.1. The lower limit of quantification (LOQ) was 0.1 µg/mL. Values below the LOQ were set to 0.05 µg/mL for the statistical analysis.

### 2.8. Statistical Analysis

IBM SPSS Statistics 25 software was used for calculations and graph preparation. Spearman rank correlation was used to measure any statistically significant association between the variables

The effect of time lapse, study group allocation, and the interaction of these variables with clindamycin concentrations and post-operative pain levels were evaluated using the analysis of variance in the mixed model (with repeated measures). Statistically significant interaction of the variables was examined by the analysis of the simple main effects. The size of the effect was measured using the Eta-squared value. In case of a lack of sphericity, Greenhouse–Geisser correction was applied.

The chi-square test was used to check whether the compared groups were equipotential and assess the presence of statistically significant correlations between the nominal variables. The Eta coefficient was used to evaluate the correlations between the quantitative and nominal variables. For nominal variables, the strength of association was measured using Cramer’s V.

Mean, median, standard deviation, minimum, maximum, and the first and third quartiles were used for the analysis. A *p*-value of 0.05 or less was considered statistically significant.

## 3. Results

### 3.1. Patient Characteristics

**Figure 1:**
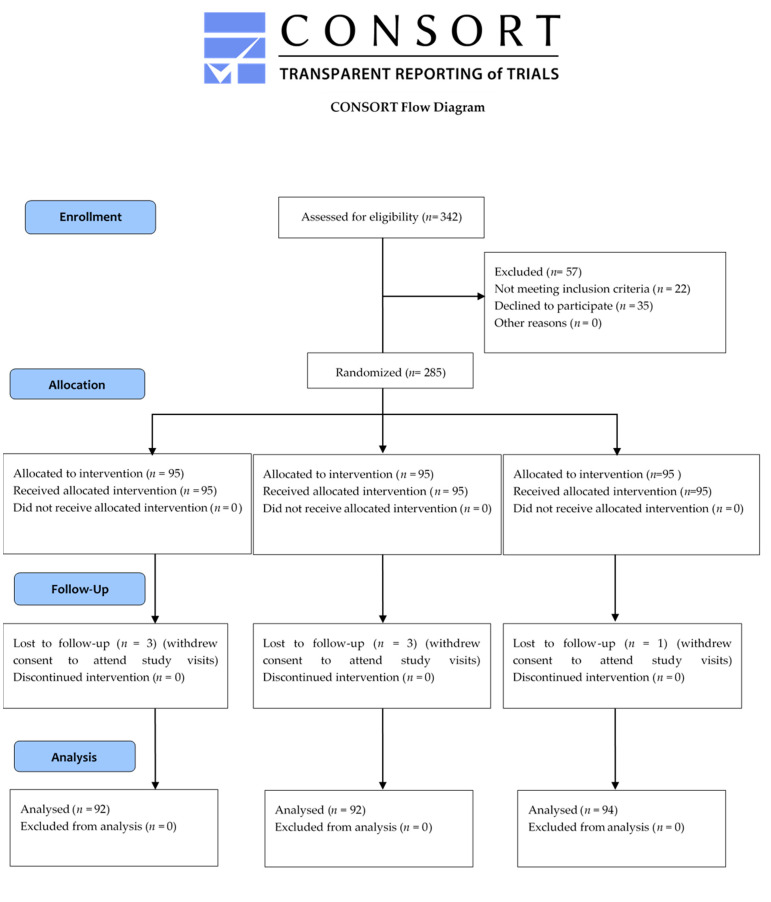
Consolidated Standards of Reporting Trials (CONSORT) flowchart depicting the study design and execution.

The sample size calculation was based on the data obtained from our pilot study, in which the infection rate was evaluated based on the results of 20 patients who received conventional antimicrobial treatment (300 mg of clindamycin hydrochloride every 8 h for 5 days). Assuming a two-tailed alpha error of 0.05, a statistical power of 90%, a mean of 6.5 points with a standard deviation of 3, expecting to detect a clinically significant difference of 2 points, 90 patients were required for each treatment arm. Assuming a 5% withdrawal rate, 95 patients per treatment arm were selected. The research was conducted in a group of 278 patients, since 6 patients were lost to follow-up; 92 (33.1%), 92 (33.1%), and 94 (33.8%) patients were included in groups I, II, and III, respectively, as presented in the Consolidated Standards of Reporting Trials (CONSORT) flowchart (Figure 1).

The comparison groups were equinumerous (χ^2^(2) = 0.03, *p* = 0.99) and evenly split considering sex and body mass index (BMI) (Table 1).

Statistically significant differences in BMI were observed in the three groups; however, the strength of the effect was low. The BMI of the patients receiving a higher dose of clindamycin (600 mg) was slightly lower than that of the patients in the second group (*p* = 0.02). There were no statistically significant differences in age between the groups.

### 3.2. Influence of Treatment on the Post-Operative Inflammatory Parameters

Considering trismus, statistically significant differences were observed for each time frame, with the exclusion of grade 1 on the third day. Grades 2 and 3 trismus on the first and third follow-up visits were observed in a larger number of patients from group III compared to the other groups. Moreover, a smaller number of patients from group III were diagnosed with grade 0 trismus on the seventh day, while a contrary observation was noted with respect to grade 1, with only participants from this group developing grade 1 trismus on the same day.

The largest effect size, measured using Cramer’s V coefficient, occurred once for grade 0 on the seventh day. The descriptive statistics are presented in Table 2.

Considering facial edema, the greatest differences were observed again for grade 0 on the seventh day, on which grade 0 edema was observed in a significantly smaller number of participants in group III, whereas the opposite was true for grade 1. It is also worth noting that at the first follow-up visit, grade 3 edema was diagnosed in 44.7% of the patients from the group receiving 600 mg of clindamycin compared to 10.9% in the other two groups, as shown in Table 3.

The results for lymphadenopathy were in conformity with previous findings, with the strongest association detected on the seventh day for grade 1 lymphadenopathy in group III; the percentage was lowest, at 24.5 to 76.1%, for the participants from group I (Table 4).

The conclusions are similar in the case of dysphagia: the strongest association occurred on the seventh day for grade 5, with the lowest percentage of patients able to swallow normally in group III. A similar observation was noted for grade 4 and the opposite for grade 3 in the same time frame. Descriptive statistics are presented in Table 5.

Considering elevated temperature and dry socket, the strongest association was found at the second follow-up visit, with group III demonstrating distinctive variables. Interestingly, dry socket did not occur in group I, and only one patient from group II was diagnosed with this condition. The statistical results are summarized in Table 6.

### 3.3. Influence of Treatment on Pain Intensity

The statistically significant difference between each group in terms of intensity of post-operative pain was especially relevant in the first, third, and seventh day (strong effect measured with eta-squared). Pairwise comparisons revealed that at 60 min following the administration of the first dose, the level of pain in group I was lower than in groups II (*p* = 0.01) and III (*p* < 0.001). In the case of other time frames, the groups differed significantly from one another (*p* < 0.001). Interestingly, the lowest values were observed among patients in group I, while the highest values were observed in group III, as shown in Table 7.

In addition, the need for rescue drugs presumably confirms the above-mentioned findings, since this result was significant for group III (600 mg of clindamycin), as presented in Table 8.

### 3.4. Adverse Effects

Stomach and abdominal pain, nausea, and vomiting were the most common adverse effects reported by patients. Interestingly, there were no reports of any adverse effects in group I and only four cases of stomach pain in group II. The situation was much worse in the patients belonging to group III, for which 23 cases of stomach and abdominal pain were reported, along with 7 cases of nausea, 3 cases of vomiting, and 1 case of chest pain.

### 3.5. Clindamycin Concentrations in Saliva

The achieved effect size indicated significant differences in clindamycin concentrations between the study groups. The highest value was observed in group III, which was expected. Each cluster differed statistically significantly from one another (*p* < 0.001), with the only exception being group I in relation to group II on the seventh day (*p* = 0.29), as presented in Table 9. The power of the statistical tests was set at 100%.

Subsequently, the statistical association between clindamycin concentration and pain intensity was examined; however, no significant association was observed.

Table 10 presents the results of the correlation analysis for clindamycin concentration, age, and BMI. The higher the BMI, the lower the concentration of the drug in saliva, with the highest strength in participants receiving 150 mg of clindamycin, while the older the patient, the lower the concentration of the drug on the first and third days.

**Figure 2:**
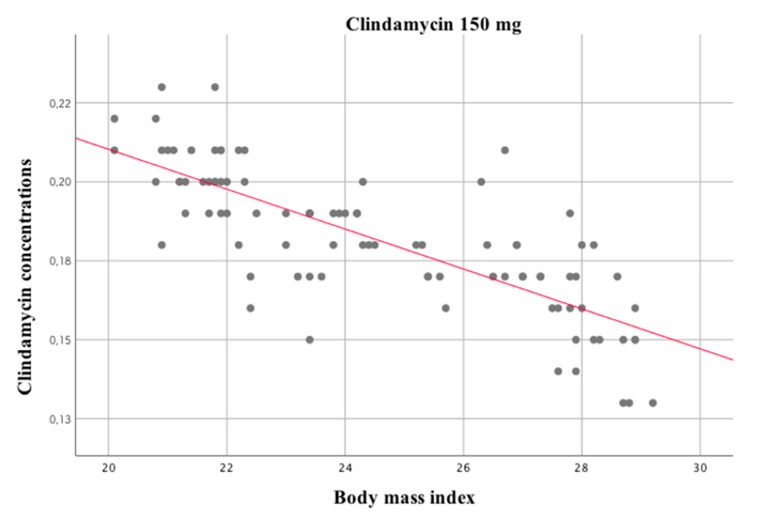
Correlation between the body mass index of patients receiving 150 mg of clindamycin and its concentration on the third day.

The strongest relationship was observed on the third day post-surgery, that is, between the BMI of patients belonging to group I, as presented in Figure 2.

In the next step, the associations between the sex of the participants in each group, pain levels, and clindamycin concentrations were examined; however, no association was found.

By conducting an analysis of the simple main effects, we observed a higher concentration of clindamycin in women than in men (*p* < 0.01) on the first and third days in group III.

Lastly, the Eta coefficient was used to assess the statistical association between the grades of the parameters of post-operative inflammation and clindamycin concentration in saliva based on individual time frames for the three groups. In each studied cluster broken down into a particular time frame, the grade of each feature (0–3 or 0–5) did not exhibit a statistically significant association with clindamycin concentration. The value of the coefficient (which can vary between 0 and 1) with respect to the association between the concentration in the three time frames and the grade of each parameter was calculated as zero, which indicated that there was no relationship between clindamycin concentration in saliva and degree of post-operative inflammation.

## 4. Discussion

Despite many controversies surrounding antibiotic therapy in the case of third molar surgery, the debate is still ongoing, with emphasis on ascertaining the specific indications, as some authors highlight the risks of adverse reactions and the development of resistant bacteria [18,19]. Although some studies advocate no potential benefit [20,21], the prophylactic use of antibiotics is still in effect, since, in our opinion, it drastically reduces post-operative complications. The results of research that compared infection rates with and without antibiotic administration and did not observe a decrease in infection rate [22,23] seem dubious; moreover, these results could be due to differences in qualifying and reporting the effects as complications or infections. As suggested by Osborn et al. [22], the rate of post-operative infections is higher for impacted mandibular molars that require osteotomy, which is especially true for immunocompromised patients and for patients who do not comply with and follow the post-operative procedures outlined by the surgeon. Perhaps surprisingly, the percentages of such patients experienced in the daily lives of oral surgeons are quite high.

Our study demonstrates that a post-operative course of clindamycin is beneficial in terms of alleviating pain and decreasing complications and infection, defined here as the incidence of dry socket, especially with lower doses administered over shorter time periods. We conducted a clinical trial under the usual working conditions of two public hospitals in different countries and the results were consistent. The study was carefully designed to avoid the risk of bias as far as possible, and to further limit such risks, the classification of the resulting inflammatory and infectious complications was conducted based on the clinical symptoms described in other studies [7,22,24]. One of the limitations of this study was the lack of a placebo group; however, it was designed with the intent-to-treat approach and, in addition, over more than 15 years of experience with antibiotic prophylaxis on a daily basis in a variety of cases, contrary to some authors’ opinions [19,23,25,26], the usage of antibiotics has rarely been deemed preemptive. Even though the above-mentioned research—for example, the study of Rohit and Praveen Reddy [26]—failed to prove the advantages of routine post-operative use of antibiotics in third molar surgery, this is mainly due to the fact that in most cases the antibiotic used was either amoxicillin alone or combined with metronidazole. Our clinical experience confirms that this combination fails to bring about the long-term desired effect.

We do take into consideration the fact that a long duration and multiple courses of antibiotics are associated with bacterial resistance; however, our research clearly demonstrates that a course of 150 mg clindamycin every 8 h for 5 days not only alleviates pain but also decreases the severity of post-operative sequelae, providing relief for patients. A similar finding was reported by conducted by Kaczmarzyk et al. with 600 mg clindamycin administered preoperatively [15]; however, only one arm of this research could be compared with our design, and the obtained results were not as promising, with surprisingly poor analgesic effects. As previously stated, available clinical trials that focus only on clindamycin in third molar surgery are scarce. In a systematic review by Lodi et al. [2], whose objective was to determine the effect of systemic antibiotic prophylaxis on the prevention of complications following tooth extractions, out of 21 studies in which antibiotics were administered orally, only 3 involved clindamycin, with one being conducted in 1972.

Although clindamycin is considered to be well tolerated and safe, its administration may cause unpleasant adverse effects, especially in the gastrointestinal tract, with diarrhea being the most commonly reported side effect [27]. Although, according to different studies, its incidence varies from 2 to 20% [28], we did not observe this effect during our study. However, we observed other symptoms, such as stomach and abdominal pain, in which the incidence increased with an increase in the doses of clindamycin administered. The most adverse effects were reported by patients in group III, whereas no adverse effects were noted in group I, which may be another argument in favor of smaller doses administered over shorter time periods, especially since our research proved that there is no association between clindamycin concentration and degree of post-operative inflammation.

Every tissue injury triggers the immune system and results in an inflammatory response that activates the pain pathways. Even though pain is handled by the nervous system, the immune cells and glia interact with neurons, which results in the remodeling of pain levels and the alteration of pain from acute to chronic pain [29]. Antibiotics aid the immune system in battling infection and subsequently dismiss the cause of inflammation and pain; however, some studies [30,31] state that such an action may be attributed to non-microbial activity. Although the antimicrobial effect of clindamycin is indisputable, some studies [32,33] have demonstrated that clindamycin may inhibit the production of tumor necrosis factor-alpha (TNFα), interleukin-1(IL-1), and cytokines. Therefore, its non-antimicrobial immunomodulatory influence should be emphasized. Our study also aimed to investigate the effect of clindamycin on pain to determine whether a course of this drug could facilitate positive outcomes. Taking into account the relatively short terminal half-life of this lincosamide [34], its long-lasting influence on pain levels is quite impressive. Interestingly, our research shows that lower pain levels were observed in patients receiving lower doses over shorter periods of time, which would at least partially explain the prolonged effect, even though our results contradict the studies by Olusanya et al. [23] and Kaczmarzyk et al. [15], who stated that there was no significant difference in post-operative pain between the group who received antibiotics and those who did not. The prolonged influence on pain could be attributed to the concentration of clindamycin in the acidic environment of neutrophilic lysosomes [35,36], which, in turn, plays a major role in inducing symptoms of inflammatory response through the production of various molecules. We believe that further research is warranted to completely understand these mechanisms and examine the role of clindamycin in inhibiting concentrations of TNFα in humans to pave the way for other potential applications in the treatment of non-infectious diseases.

## 5. Conclusions

Our study suggests that lower doses of clindamycin administered over shorter time periods are beneficial in relieving pain and reducing post-operative complications following third molar surgery; hence, clinicians should consider prescribing clindamycin at a dose of 150 mg in cases of bony impacted third molars.

## Figures and Tables

**Table 1:** Patient characteristics.

Variable		Clindamycin Dose						Statistical Test Results *
		150 mg		300 mg		600 mg		
		*n*	%	*n*	%	*n*	%	
Sex	Female	50	54.3	44	47.8	40	42.6	χ^2^(2) = 2.6; *p* = 0.27
	Male	42	45.7	48	52.2	54	57.4	
BMI	Normal weight	53	57.6	43	46.7	51	54.3	χ^2^(2) = 2.29; *p* = 0.32
	Overweight	39	42.4	49	53.3	43	45.7	

BMI, body mass index. * Chi-square.

**Table 2:** Trismus in the study groups.

Variable		Clindamycin Dose						Statistical Test Results *
		150 mg		300 mg		600 mg		
		*n*	%	*n*	%	*n*	%	
Trismus, day 1	Grade 0	11	12	12	13	0	0	χ^2^(2) = 12.88; *p* = 0.002; Vcr = 0.22
	Grade 1	70	76.1	69	75	34	36.2	χ^2^(2) = 41.06; *p* < 0.001; Vcr = 0.38
	Grade 2	11	12	8	8.7	44	46.8	χ^2^(2) = 47.53; *p* < 0.001; Vcr = 0.41
	Grade 3	0	0	3	3.3	16	17	χ^2^(2) = 23.91; *p* < 0.001; Vcr = 0.29
Trismus, day 3	Grade 0	40	43.5	23	25	0	0	χ^2^(2) = 50.58; *p* < 0.001; Vcr = 0.43
	Grade 1	51	55.4	61	66.3	50	53.2	χ^2^(2) = 3.74; p = 0.15
	Grade 2	1	1.1	5	5.4	44	46.8	χ^2^(2) = 80.58; *p* < 0.001; Vcr = 0.54
	Grade 3	0	0	2	2.2	0	0	χ^2^(2) = 4.07; *p* = 0.13
Trismus, day 7	Grade 0	92	100	92	100	45	47.9	χ^2^(2) = 116.44; *p* < 0.001; Vcr = 0.65
	Grade 1	0	0	0	0	47	50	χ^2^(2) = 110.71; *p* < 0.001; Vcr = 0.63
	Grade 2	0	0	0	0	0	0	-
	Grade 3	0	0	0	0	0	0	-

* Chi-square, Cramer’s V coefficient.

**Table 3:** Facial edema in the study groups.

Variable		Clindamycin Dose						Statistical Test Results *
		150 mg		300 mg		600 mg		
		*n*	%	*n*	%	*n*	%	
Facial edema, day 1	Grade 0	1	1.1	1	1.1	0	0	χ^2^(2) = 1.03; *p* = 0.6
	Grade 1	13	14.1	11	12	0	0	χ^2^(2) = 13.7; *p* = 0.001; Vcr = 0.22
	Grade 2	67	72.8	69	75	52	55.3	χ^2^(2) = 9.92; *p* = 0.007; Vcr = 0.19
	Grade 3	10	10.9	10	10.9	42	44.7	χ^2^(2) = 41.05; *p* < 0.001; Vcr = 0.38
Facial edema, day 3	Grade 0	9	9.8	9	9.8	0	0	χ^2^(2) = 9.83; *p* = 0.007; Vcr = 0.19
	Grade 1	76	82.6	57	62	1	1.1	χ^2^(2) = 134.25; *p* < 0.001; Vcr = 0.7
	Grade 2	7	7.6	26	28.3	53	56.4	χ^2^(2) = 52.23; *p* < 0.001; Vcr = 0.43
	Grade 3	0	0	0	0	40	42.6	χ^2^(2) = 91.46; *p* < 0.001; Vcr = 0.57
Facial edema, day 7	Grade 0	92	100	90	97.8	46	48.9	χ^2^(2) = 105.5; *p* < 0.001; Vcr = 0.62
	Grade 1	0	0	2	2.2	45	47.9	χ^2^(2) = 97.09; *p* < 0.001; Vcr = 0.59
	Grade 2	0	0	0	0	3	3.2	χ^2^(2) = 5.94; *p* = 0.051
	Grade 3	0	0	0	0	0	0	-

* Chi-square, Cramer’s V coefficient.

**Table 4:** Lyphmadenopathy in the study groups.

Variable		Clindamycin Dose						Statistical Test Results *
		150 mg		300 mg		600 mg		
		*n*	%	*n*	%	*n*	%	
Lymphadenopathy, day 1	Grade 0	0	0	0	0	0	0	-
	Grade 1	14	15.2	14	15.2	0	0	χ^2^(2) = 15.91; *p* < 0.001; Vcr = 0.24
	Grade 2	66	71.7	65	70.7	50	53.2	χ^2^(2) = 8.9; *p* = 0.01; Vcr = 0.18
	Grade 3	12	13	13	14.1	44	46.8	χ^2^(2) = 36.83; *p* < 0.001; Vcr = 0.36
Lymphadenopathy, day 3	Grade 0	0	0	0	0	0	0	χ^2^(2) = 9.83; *p* = 0.007; Vcr = 0.19
	Grade 1	67	72.8	14	15.2	33	35.1	χ^2^(2) = 65.15; *p* < 0.001; Vcr = 0.48
	Grade 2	25	27.2	65	70.7	19	20.2	χ^2^(2) = 57.98; *p* < 0.001; Vcr = 0.46
	Grade 3	0	0	13	14.1	42	44.7	χ^2^(2) = 61.53; *p* < 0.001; Vcr = 0.47
Lymphadenopathy, day 7	Grade 0	22	23.9	34	37	29	30.9	χ^2^(2) = 3.69; *p* = 0.16
	Grade 1	70	76.1	59	64.1	23	24.5	χ^2^(2) = 96.84; *p* < 0.001; Vcr = 0.59
	Grade 2	0	0	0	0	42	44.7	χ^2^(2) = 5.94; *p* = 0.051
	Grade 3	0	0	0	0	0	0	-

* Chi-square, Cramer’s V coefficient.

**Table 5:** Dysphagia in the study groups.

Variable		Clindamycin Dose						Statistical Test Results *
		150 mg		300 mg		600 mg		
		*n*	%	*n*	%	*n*	%	
Dysphagia, day 1	Grade 0	0	0	0	0	0	0	-
	Grade 1	0	0	0	0	0	0	-
	Grade 2	0	0	1	1.1	48	51.1	χ^2^(2) = 109.41; *p* < 0.001; Vcr = 0.63
	Grade 3	25	27.2	17	18.5	44	46.8	χ^2^(2) = 18.38; *p* < 0.001; Vcr = 0.26
	Grade 4	69	75	62	67.4	2	2.1	χ^2^(2) = 120; *p* < 0.001; Vcr = 0.66
	Grade 5	1	1.1	14	15.2	0	0	χ^2^(2) = 26.09; *p* < 0.001; Vcr = 0.31
Dysphagia, day 3	Grade 0	0	0	0	0	0	0	-
	Grade 1	0	0	0	0	0	0	-
	Grade 2	0	0	3	3.3	44	46.8	χ^2^(2) = 90.74; *p* < 0.001; Vcr = 0.23
	Grade 3	11	12	20	21.7	3	3.2	χ^2^(2) = 14.91; *p* = 0.001; Vcr = 0.47
	Grade 4	48	52.2	41	44.6	44	46.8	χ^2^(2) = 1.13; *p* = 0.57
	Grade 5	33	35.9	28	30.4	2	2.1	χ^2^(2) = 34.94; *p* < 0.001; Vcr = 0.36
Dysphagia, day 7	Grade 0	0	0	0	0	0	0	-
	Grade 1	0	0	0	0	0	0	-
	Grade 2	0	0	0	0	0	0	-
	Grade 3	0	0	0	0	49	52.1	χ^2^(2) = 116.44; *p* < 0.001; Vcr = 0.65
	Grade 4	0	0	0	0	13	13.8	χ^2^(2) = 26.7; *p* < 0.001; Vcr = 0.31
	Grade 5	92	100	92	100	32	34	χ^2^(2) = 156.2; *p* < 0.001; Vcr = 0.75

* Chi-square, Cramer’s V coefficient.

**Table 6:** Occurrence of elevated temperature and local infection.

Variable		Clindamycin Dose						Statistical Test Results *
		150 mg		300 mg		600 mg		
		*n*	%	*n*	%	*n*	%	
Day 1	Temperature >37.5 °C	0	0	3	3.3	43	45.7	χ^2^(2) = 88.04; *p* < 0.001; Vcr = 0.56
	Dry socket	0	0	0	0	0	0	-
Day 3	Temperature >37.5 °C	0	0	1	1.1	43	45.7	χ^2^(2) = 95.46; *p* < 0.001; Vcr = 0.59
	Dry socket	0	0	1	1.1	16	17	χ^2^(2) = 29.52; *p* < 0.001; Vcr = 0.33
Day 7	Temperature >37.5 °C	0	0	0	0	0	0	-
	Dry socket	0	0	0	0	11	4	χ^2^(2) = 22.42; *p* < 0.001; Vcr = 0.28

* Chi-square, Cramer’s V coefficient.

**Table 7:** Descriptive statistics of post-operative pain in the study groups.

Post-Operative Pain	Dose of Clindamycin	M	Me	SD	Min	Max	Q1	Q3	Statistical Test Results
60 min after administration	150 mg	2.9	2.8	1.05	1	6	2.3	3.5	F(2;275) = 12.36; *p* < 0.001; eta^2^ = 0.08
	300 mg	3.34	3.1	1.04	1	6.1	2.7	4	
	600 mg	3.63	3.6	0.9	2	7	3	4	
Day 1	150 mg	1.68	1.45	0.84	0.1	4.2	1	2.3	F(2;275) = 128.99; *p* < 0.001; eta^2^ = 0.48
	300 mg	2.22	2	0.76	0.4	4.2	1.8	2.78	
	600 mg	4.3	3.65	1.68	1.5	8	2.9	5.93	
Day 3	150 mg	1.2	1	0.59	0	4	1	1.15	F(2;275) = 138.24; *p* < 0.001; eta^2^ = 0.5
	300 mg	1.44	1.3	0.56	0.1	3.5	1	1.8	
	600 mg	3.77	2.95	1.84	1	7.5	2.1	5.63	
Day 7	150 mg	0.1	0	0.25	0	1	0	0	F(2;275) = 159.04; *p* < 0.001; eta^2^ = 0.54
	300 mg	0.54	0.5	0.42	0	1.5	0	1	
	600 mg	2	1.8	1.22	0.4	5.7	1	2.5	

M, mean; Me, median; SD, standard deviation; Min, minimum; Max, maximum, Q1, first quartile; Q3, third quartile.

**Table 8:** Use of rescue drug in the study groups.

Variable	Clindamycin Dose						Statistical Test Results *
	150 mg		300 mg		600 mg		
	*n*	%	*n*	%	*n*	%	
Use of rescue drug	2	2.2	0	0	23	24.5	χ^2^(2) = 41.82; *p* < 0.001; Vcr = 0.39

* Chi-square, Cramer’s V coefficient.

**Table 9:** Concentrations of clindamycin in the study groups based on individual time frames.

Clindamycin Concentrations	Dose of Clindamycin	M	Me	SD	Min	Max	Q1	Q3	Statistical Test Results
Day 1 (24 h)	150 mg	0.17	0.17	0.02	0.12	0.21	0.15	0.18	F(2;275) = 1514.4; *p* < 0.001; eta^2^ = 0.92
	300 mg	0.37	0.37	0.06	0.21	0.51	0.33	0.4	
	600 mg	0.74	0.71	0.11	0.59	1.12	0.69	0.75	
Day 3 (72 h)	150 mg	0.18	0.18	0.02	0.13	0.23	0.17	0.2	F(2;275) = 1223.1; *p* < 0.001; eta^2^ = 0.9
	300 mg	0.39	0.4	0.06	0.24	0.54	0.37	0.43	
	600 mg	0.79	0.75	0.13	0.62	1.24	0.73	0.8	
Day 7 (168 h)	150 mg	0.13	0.08	0.17	0.05	0.9	0.07	0.09	F(2;275) = 42.43; *p* < 0.001; eta^2^ = 0.24
	300 mg	0.15	0.16	0.03	0.09	0.24	0.14	0.17	
	600 mg	0.26	0.25	0.06	0.19	0.47	0.23	0.3	

M, mean; Me, median; SD, standard deviation; Min, minimum; Max, maximum, Q1, first quartile; Q3, third quartile.

**Table 10:** Relationship between BMI, age, and clindamycin concentration in each group.

Clindamycin Concentration	BMI			Age		
	150 mg	300 mg	600 mg	150 mg	300 mg	600 mg
Day 1	r_s_ = −0.79; *p* < 0.001	r_s_ = −0.32; *p* = 0.002	r_s_ = −0.41; *p* < 0.001	r_s_ = −0.22; *p* = 0.03	r_s_ = −0.06; *p* = 0.57	r_s_ = −0.27; *p* = 0.007
Day 3	r_s_ = −0.8; *p* < 0.001	r_s_ = −0.33; *p* = 0.001	r_s_ = −0.46; *p* < 0.001	r_s_ = −0.22; *p* = 0.04	r_s_ = −0.09; *p* = 0.41	r_s_ = −0.31; *p* = 0.002
Day 7	r_s_ = −0.5; *p* < 0.001	r_s_ = −0.31; *p* = 0.002	r_s_ = −0.33; *p* = 0.001	r_s_ = −0.04; *p* = 0.69	r_s_ = −0.05; *p* = 0.64	r_s_ = −0.14; *p* = 0.19

BMI, body mass index; M, mean; Me, median; SD, standard deviation; Min, minimum; Max, maximum, Q1, first quartile; Q3, third quartile.

## Data Availability

The data presented in this study are available upon request from the corresponding author. The data are not publicly available since certain data are subject to further research.
